# Biological response of nonhuman primates to controlled levels of acute blood loss

**DOI:** 10.3389/fimmu.2023.1286632

**Published:** 2024-01-10

**Authors:** Juhye Roh, Eun Mi Park, Haneulnari Lee, Jeong Ho Hwang, Hyung-Sun Kim, Jinyoung Park, Hee Jung Kang

**Affiliations:** ^1^ Department of Laboratory Medicine, Hallym University College of Medicine and Hallym University Sacred Heart Hospital, Anyang, Republic of Korea; ^2^ Animal Model Research Group, Jeonbuk Branch Institute, Korea Institute of Toxicology, Jeongeup, Republic of Korea; ^3^ Department of Psychology and Neuroscience, Duke University, Durham, NC, United States

**Keywords:** biological response in hemorrhage, controlled blood loss, immune response, nonhuman primate, xenotransfusion

## Abstract

**Introduction:**

The global shortage of human blood for medical use has prompted the development of alternative blood sources. Nonhuman primates (NHPs) are commonly used owing to their physiological similarities to humans. The objective of the current study was to establish a controlled-blood-loss model in NHPs to explore their clinical and biological responses.

**Methods:**

Blood was sequentially withdrawn from 10 cynomolgus monkeys (10, 14, 18, 22, and 25% of the total blood volume); their vital signs were monitored, and blood parameters were serially analyzed. Humoral mediators in the blood were measured using flow cytometry and enzyme-linked immunosorbent assays.

**Results:**

In NHPs subjects to 25% blood loss and presenting with related clinical symptoms, the systolic blood pressure ratio on day 0 after bleeding was significantly lower than that of the animals from the other groups (median: 0.65 vs. 0.88, *P* = 0.0444). Red blood cell counts from day 0–14 and hematocrit levels from day 0–7 were markedly decreased relative to the baseline (*P* < 0.01). These parameters showed a direct correlation with the extent of blood loss. The levels of creatine phosphokinase, aspartate aminotransferase, and alanine aminotransferase exhibited increases in response to blood loss and had a stronger correlation with the hemoglobin ratio than the volume of blood loss. The levels of C3a and C4a, as well as interleukin (IL)-1α and IL-15, displayed a strong correlation, with no apparent association with blood loss.

**Conclusion:**

The findings of the present study showed that only NHPs with 25% blood loss exhibited clinical decompensation and significant systolic blood pressure reduction without fatalities, suggesting that this level of blood loss is suitable for evaluating blood transfusion efficacy or other treatments in NHP models. In addition, the ratio of hemoglobin may serve as a more dependable marker for predicting clinical status than the actual volume of blood loss. Thus, our study could serve as a basis for future xenotransfusion research and to predict biological responses to massive blood loss in humans where controlled experiments cannot be ethically performed.

## 1 Introduction

Blood transfusions are an important aspect of substitution therapy, although the availability of blood products is often limited in low- and middle-income countries (1). According to data from 2017, 119 of the 195 analyzed countries (61%) had an insufficient blood supply. Lack of adequate blood supply has been shown to contribute to preventable mortality and morbidity in several low- and middle-income countries (2). In developed countries, the low birth rate and aging of the baby-boom generation (3) could result in a decrease in the blood donor population and eventually a shortage of blood supply.

Research on blood substitutes is actively pursued to overcome blood shortages. The objective of this field of research is to develop alternative products or technologies capable of replacing human blood in blood transfusions and an alternative means of resuscitation and oxygen delivery. Hemoglobin-based oxygen carriers, perfluorocarbon-based oxygen carriers, and synthetic blood components have been developed, and the application of stem cell-derived red blood cells (RBCs) has been explored (4, 5). Alternatively, the use of animal-derived RBCs could expand the available blood supply. Pigs have been identified as potential organ and blood-cell donors for human transplantation (xenotransplantation) and transfusion (xenotransfusion) (6). Although the lifespan of pig RBCs is lower than that of human RBCs, the size and number of pig and human RBCs are comparable (7).

Nonhuman primates (NHPs), such as monkeys, have been primarily used before human clinical trials owing to similarity in their anatomical structure and physiological characteristics to those of humans (8). Previous hemorrhage animal models using pigs or dogs include the chronic anemia and acute blood loss models (9, 10). Xenogeneic blood products would primarily be needed in emergency situations for short-term resuscitation in clinical practice. Therefore, we developed an acute blood-loss model in NHPs. We attempted to determine the point of blood loss that is clinically meaningful but not detrimental to NHPs. Hence, dose escalation was performed in a stepwise manner, beginning with a bleeding condition of 10%. To distinguish between transfusion and hemorrhage-related biological responses, we investigated hemorrhage-induced biological responses.

The main objective of the current study was to explore clinical and biological responses after bleeding in an NHP model. To understand the sequences of various biological responses in NHPs after blood loss, we analyzed the expression of hematological markers, biochemical markers, inflammatory cytokines, and complement activation products in serially drawn blood samples from NHPs. Additionally, vital signs were monitored to determine the clinical status of the NHPs.

## 2 Materials and methods

### 2.1 Study design

We used 10 cynomolgus monkeys (*Macaca fascicularis;* Nafovanny, Dong Nai, Vietnam) with the authorization of the Korea Institute of Toxicology Institutional Animal Care and Use Committee (IAC-22-01-0342-0037).

**Figure 1:**
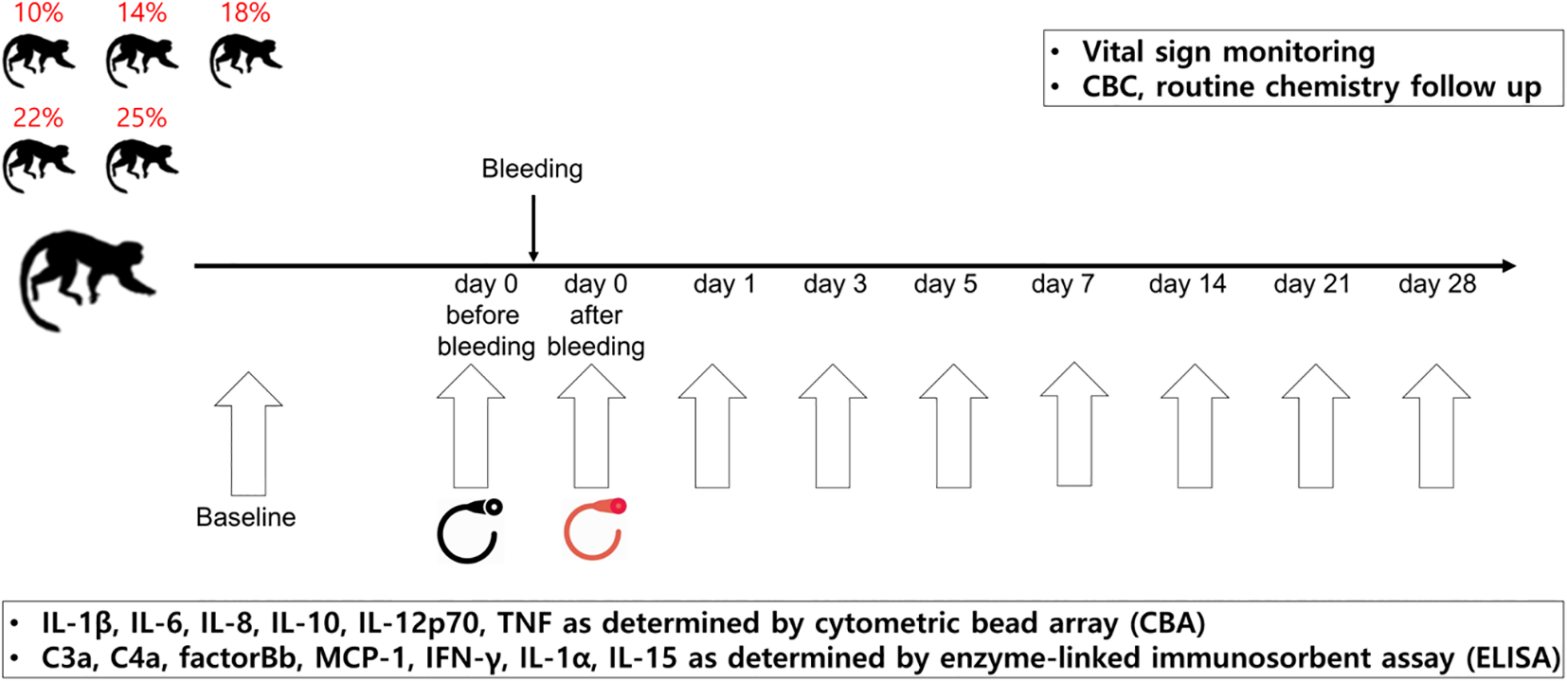
Study schema. The NHP bleeding model was developed using ten cynomolgus monkeys. The NHPs were bled by 10, 14, 18, 22, or 25% of their total blood volume. From the day of blood loss intervention, vital signs, general symptoms, and changes in appearance and behavior were monitored daily for three weeks. The blood parameters of the NHPs were analyzed on each day (day -14, 0 before bleeding, 0 after bleeding, 1, 3, 5, 7, 14, 21, and 28). Two NHPs were assigned per condition. NHP, nonhuman primate; CBC, complete blood count.

A schematic illustration of the study is shown in **Figure 1**. To develop the NHP bleeding model without lethality in experimental animals, a 10% blood loss was initially performed, which was gradually increased to 14, 18, 22, and 25% of the total blood volume, in two NHPs for each bleeding condition. The total blood volume of the NHP was calculated to be 65 mL per/kg body weight (11). The characteristics of each NHP by bleeding condition are described in **Table 1**. On the day of the acute blood loss intervention, NHPs were secured to a primate chair. The femoral vein was selected as the blood collection route, and blood collection was performed using the femoral triangle of the inguinal-proximal leg as an access route. Syringes were used to measure the target blood volume. The blood collection site was disinfected with 70% alcohol, and the planned volume of blood was drawn from the cephalic or femoral vein using a 21–23G needle while monitoring blood pressure. Vital signs and general symptoms, including mortality, morbidity, and changes in appearance and behavior, were observed daily for three weeks from the day of blood loss. The blood parameters of the NHPs were analyzed on days -14 (as baselines, at least 2 weeks before the intervention), 0 (before the bleeding intervention, D0BB), 0 after bleeding (immediately after the bleeding intervention, D0AB), 1, 3, 5, 7, 14, 21, and 28 (D1, D3, D5, D7, D14, D21, and D28, respectively).

**Table 1:** Characteristics of the nonhuman primates in this study.

Bleeding condition	Unit number	Sex	Weight (g)	Age
10%	1M1	Male	2585	3yr 3mo
10%	1F1	Female	2193	2yr 9mo
14%	2M1	Male	2542	3yr 3mo
14%	2F1	Female	2262	2yr 11mo
18%	3M1	Male	2211	3yr 3mo
18%	3F1	Female	2246	2yr 9mo
22%	4M1	Male	2531	3yr 3mo
22%	4F1	Female	2702	3yr 1mo
25%	5M1	Male	2644	3yr 5mo
25%	5F1	Female	2640	2yr 10mo

### 2.2 Measurement of the levels of biomarkers

Hematology tests were performed to determine the levels of total white blood cells (WBCs), total RBCs, hemoglobin, hematocrit, mean corpuscular volume (MCV), mean corpuscular hemoglobin concentration (MCHC), mean corpuscular hemoglobin (MCH), platelet, reticulocyte, and white blood cell differentials: neutrophils, lymphocytes, monocytes, eosinophils, and basophils. Biochemical tests were performed to estimate the levels of glucose, blood urea nitrogen (BUN), creatinine, total protein, albumin, total cholesterol, triglycerides, aspartate aminotransferase (AST), alanine aminotransferase (ALT), iron, total iron-binding capacity (TIBC), total bilirubin, alkaline phosphatase (ALP), gamma glutamyl transpeptidase (GGT), creatine phosphokinase (CK), calcium, potassium, and unsaturated iron-binding capacity (UIBC).

Using a cytometric bead array human inflammatory cytokine kit (BD Biosciences, San Diego, CA, USA), we measured plasma levels of interleukin (IL)-1β, IL-6, IL-8, IL-10, IL-12p70, and tumor necrosis factor with flow cytometry. Interferon (IFN)-γ, IL-1α, IL-15, and monocyte chemoattractant protein-1 (MCP-1) levels were measured via enzyme-linked immunosorbent assay (ELISA) using monkey IFN-γ ELISAPRO (MABTECH, Stockholm, Sweden), monkey IL-1α ELISA, monkey IL-15 ELISA (Cusabio, Wuhan, China), and monkey MCP1 ELISA kit (Abcam, Cambridge, UK), respectively. To assess the extent of complement activation, C3a,C4a and factor Bb were measured using ELISA with human C3a,C4a ELISA kit (BD Biosciences) and a Bb plus EIA kit (Quidel, San Diego, CA, USA). The detailed information is described in **Table 2**. Each measurement using ELISA or flow cytometry was performed in duplicate.

**Table 2:** Limit of detection of analytical assays for complement factors and cytokines/chemokines.

Item	Product information (Company)	Limit of detection
C3a	Human C3a ELISA kit (BD)	0.007 ng/mL
C4a	Human C4a ELISA kit (BD)	0.006 ng/mL
IFN-γ	ELISA Pro: Monkey IFN-γ (Mabtech)	1 pg/mL
MCP-1	monkey MCP1 ELISA kit (Abcam)	9.4 pg/mL
IL-1α	Monkey Interleukin 15, IL-15 ELISA Kit (Cusabio)	3.9 pg/mL
IL-15	Monkey Interleukin-1 alpha ELISA kit (Cusabio)	1.95 pg/mL
Factor Bb	MicroVue Bb Plus Fragment EIA (Quidel)	0.018 µg/mL
IL-1β	Cytometric Bead Array Human Inflammatory Cytokine Kit (BD)	7.2 pg/mL
IL-6	Cytometric Bead Array Human Inflammatory Cytokine Kit (BD)	2.5 pg/mL
IL-8	Cytometric Bead Array Human Inflammatory Cytokine Kit (BD)	3.6 pg/mL
IL-10	Cytometric Bead Array Human Inflammatory Cytokine Kit (BD)	3.3 pg/mL
IL-12p70	Cytometric Bead Array Human Inflammatory Cytokine Kit (BD)	1.9 pg/mL
TNF	Cytometric Bead Array Human Inflammatory Cytokine Kit (BD)	3.7 pg/mL

IFN-γ, interferon-γ; IL-1α, -15, -1β, -6, -8, -10, -12p70, interleukin-1α, -15, -1β, -6, -8, -10, -12p70; TNF, tumor necrosis factor; MCP-1, monocyte chemoattractant protein-1.

### 2.3 Statistical analysis

Statistical analyses were performed using MedCalc (version 22.0; Ostend, Belgium) and R (version 4.3.2; Vienna, Austria). The difference in the levels between baseline and a certain time point was analyzed using the Wilcoxon paired test and the difference between two groups using the Mann-Whitney test. Correlations between factors were analyzed using the Spearman’s correlation analysis. Statistical significance was set at *P* < 0.05 (*) and *P* < 0.01 (**).

## 3 Results

### 3.1 Biological characteristics of the NHPs at the baseline timepoint

The gender differences among the NHPs and the mean and standard deviation (SD) of baseline measurements segregated by gender, including the statistical significance (*P* value), are presented in **Table 3**. Notable gender-based variations were absent in most parameters, with the exception of RBC, hemoglobin, and hematocrit. However, the values of each measurement varied individually. At baseline, the levels of IL-1β, IL-6, IL-8, IL-10, IL-12p70, and tumor necrosis factor were below detectable thresholds.

**Table 3:** The baseline characteristics in nonhuman primate subjects.

	F (n = 5)		M (n = 5)		*P*	
	Mean	SD	Mean	SD		
Physiological parameters						
Body weight (g)	2457.20	275.38	2522.20	163.22	1.0000	
Systolic BP	123.60	11.38	125.27	8.44	1.0000	
Diastolic BP	72.47	10.59	69.27	5.80	0.8413	
Heart rate	270.93	30.49	265.60	23.27	0.8413	
Hematological parameters						
RBC (×10^6^/µL)	5.00	0.28	5.66	0.20	0.0159	*
Hemoglobin (g/dL)	11.92	0.64	13.38	0.33	0.0088	**
Hematocrit (%)	38.82	2.09	43.20	0.78	0.0079	**
MCV (fL)	77.60	2.55	76.40	1.71	0.6905	
MCH (pg)	23.82	1.32	23.68	0.50	0.6905	
MCHC (g/dL)	30.68	0.89	30.98	0.41	0.4535	
Reticulocytes (%)	1.37	0.24	0.87	0.28	0.0556	
Platelets (×10^3^/µL)	374.00	80.76	375.60	49.58	1.0000	
WBC (×10^3^/µL)	13.28	4.31	11.56	4.65	0.6004	
Neutrophils (×10^3^/µL)	9.29	5.06	6.38	3.85	0.5476	
Lymphocytes (×10^3^/µL)	3.64	3.54	4.80	1.65	0.5253	
Monocytes (×10^3^/µL)	0.236	0.065	0.276	0.089	0.4417	
Eosinophils (×10^3^/µL)	0.056	0.068	0.048	0.041	0.8273	
Basophils (×10^3^/µL)	0.018	0.011	0.020	0.014	0.8089	
Biochemical parameters						
Glucose (mg/dL)	82.38	33.70	74.00	13.04	0.8413	
Albumin (g/dL)	4.23	0.20	4.16	0.29	0.6905	
Total protein (g/dL)	7.22	0.29	7.17	0.56	1.0000	
BUN (mg/dL)	21.84	5.44	20.24	0.78	0.1508	
Creatinine (mg/dL)	0.89	0.11	0.78	0.14	0.1719	
AST (IU/L)	41.34	7.49	48.52	5.84	0.3095	
ALT (IU/L)	32.72	8.20	27.10	9.02	0.5476	
ALP (IU/L)	1012.50	296.01	1564.32	435.44	0.0952	
GGT (IU/L)	65.07	24.63	74.69	22.21	0.5476	
CK (IU/L)	555.80	483.39	336.12	22.43	0.8413	
Total bilirubin (mg/dL)	0.19	0.04	0.20	0.02	0.6742	
Total cholesterol (mg/dL)	123.60	19.73	103.80	22.95	1.0000	
Triglyceride (mg/dL)	31.28	14.75	32.70	17.94	0.3095	
Calcium (mg/dL)	9.78	0.27	9.92	0.32	0.8413	
Potassium (mmol/L)	4.38	0.56	4.91	0.80	0.3095	
Iron (µg/dL)	134.86	41.78	152.40	15.71	0.2222	
TIBC (µg/dL)	336.12	22.43	351.66	60.32	0.8413	
UIBC(µg/dL)	201.3	34.12	199.3	47.30	0.8413	
Immunological mediators						
C3a (µg/ml)	0.13	0.05	0.13	0.05	0.9161	
C4a (µg/ml)	0.34	0.22	0.23	0.07	0.7511	
Factor Bb (µg/ml)	0.80	0.21	0.91	0.22	0.5296	
IL-1α (pg/ml)	48.09	30.40	103.55	78.03	0.4206	
IL-15 (pg/ml)	20.08	29.28	24.85	25.94	0.4633	
MCP-1 (pg/ml)	47.82	10.78	70.13	48.56	0.2222	
IFN-γ (pg/ml)	3.94	2.95	3.13	1.12	1.0000	

ALP, alkaline phosphatase; ALT, alanine aminotransferase; AST, aspartate aminotransferase; BP, blood pressure; BUN, blood urea nitrogen; CK, creatine phosphokinase; D0AB, day 0 after bleeding; D0BB, day 0 before bleeding; GGT, gamma glutamyl transpeptidase; IL, interleukin; MCH, mean corpuscular hemoglobin; MCHC, mean corpuscular hemoglobin concentration; MCP-1, monocyte chemoattractant protein-1; MCV, mean corpuscular volume; RBC, red blood cell; TIBC, total iron-binding capacity; UIBC, unsaturated iron-binding capacity; WBC, white blood cell; *P < 0.05; **P < 0.01.

### 3.2 Changes in measured biological parameters over time following bleeding

#### 3.2.1 Changes in physiological parameters

**Figure 2:**
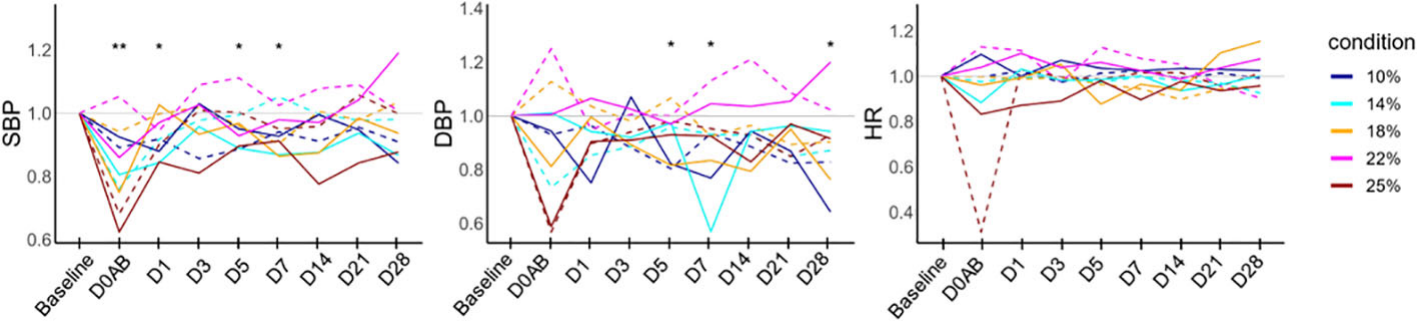
Vital sign monitoring in NHPs before and after controlled blood loss. Each group of NHPs (n=2) had 10%, 14%, 18%, 22%, or 25% of their total blood volume bled. The ratios to baseline levels were plotted: solid and dotted lines represent males and females, respectively. The levels at each time point were compared to the baseline levels, and the differences were analyzed using the Wilcoxon paired test. D0AB, day 0 after bleeding; NHP, nonhuman primates; SBP, systolic blood pressure; DBP, diastolic blood pressure; HR, heart rate; *P < 0.05; **P < 0.01.

To elucidate the temporal changes in each parameter while minimizing individual variability, the ratios of systolic and diastolic blood pressure (BP) values, as well as heart rate at each time point relative to the baseline, are graphically represented in **Figure 2**. It was observed that the systolic BP on D0AB significantly decreased when compared to the baseline (*P* < 0.01). NHPs in the 25%-blood-loss group exhibited various clinical symptoms, including pupillary dilation and, an inability to stand for ~1 h, accompanied by an immediate drop-in heart rate and blood pressure after blood loss. All NHPs were not administered crystalloid fluid regardless of symptom development, symptomatic NHPs were carefully monitored, and recovery was observed the following day.

#### 3.2.2 Variations in the levels of hematological parameters over time

**Figure 3:**
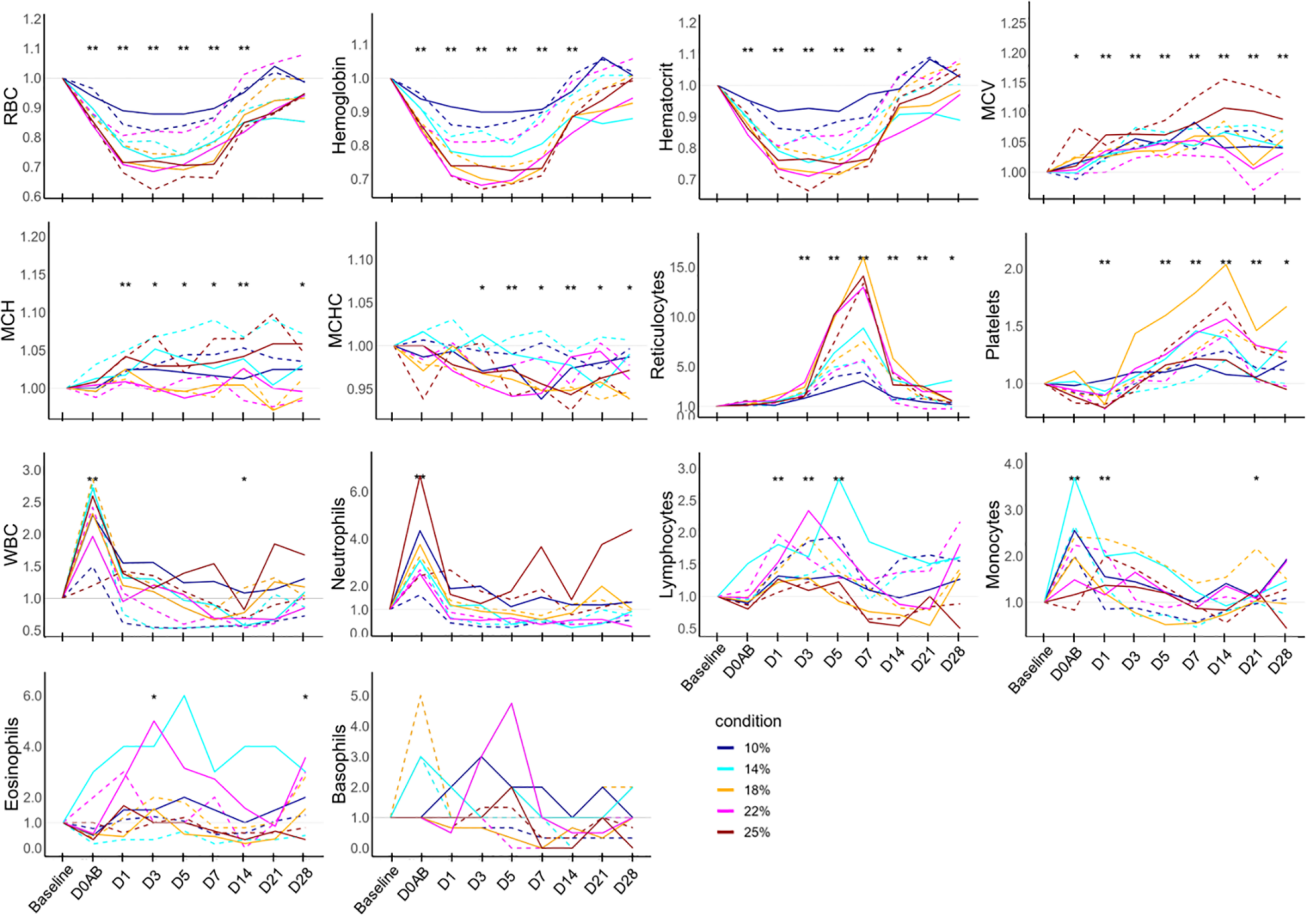
Hematology test results in NHPs before and after controlled blood loss. Each group of NHPs (n=2) had 10%, 14%, 18%, 22%, or 25% of their total blood volume bled. The ratios to baseline levels were plotted: solid and dotted lines represent males and females, respectively. The levels at each time point were compared to the baseline levels and the difference was analyzed using the Wilcoxon paired test. D0AB, day 0 after bleeding; RBC, red blood cell; MCV, mean corpuscular volume; MCH, mean corpuscular hemoglobin; MCHC, mean corpuscular hemoglobin concentration; WBC, white blood cell; *P < 0.05; **P < 0.01.

The ratios of hematological parameters at each time point compared to the baseline are depicted in **Figure 3**. On D0AB, RBC, hemoglobin, and hematocrit levels began declining, reaching their lowest on D3, followed by a gradual recovery. The levels of RBC and hemoglobin from D0AB to D14 and those of hematocrit from D0AB to D7 were significantly reduced compared to the baseline (*P* < 0.01). The MCV increased after bleeding, with values from D1 to D28 significantly exceeding the basal levels (*P* < 0.01). MCH levels on D1 and D14 were higher than baseline, whereas MCHC on D5 and D14 were lower (*P* < 0.01). The reticulocyte count began to rise on D3, peaked on D7 after blood loss, and then gradually approached the baseline values. However, even on D28, the reticulocyte count remained significantly higher than baseline (*P* < 0.05). The platelet count increased on D1, with values on D5, D7, and D14 significantly surpassing the baseline levels. The WBC, neutrophil, and monocyte counts significantly increased at D0AB but returned to the baseline levels on D1 or D3.

#### 3.2.3 Temporal dynamics in the levels of biochemical parameters

**Figure 4:**
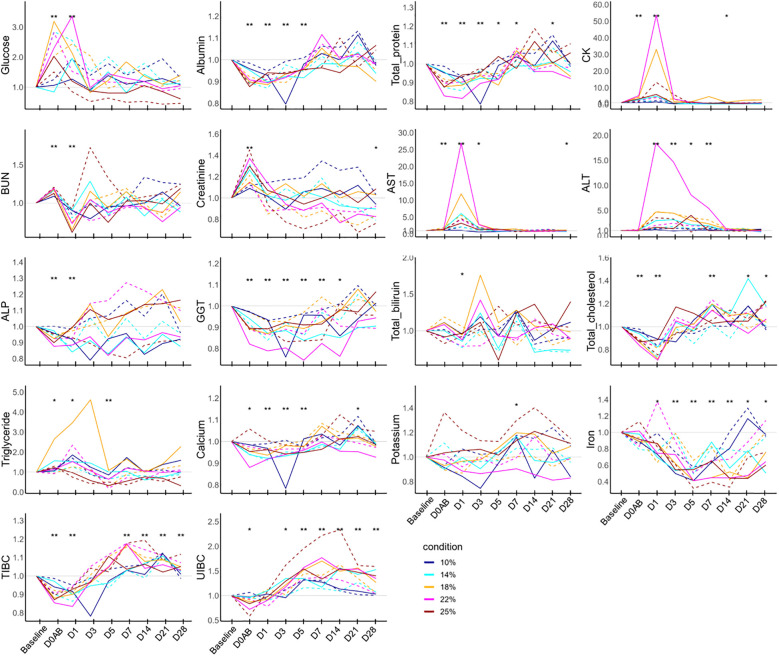
Biochemistry test results in NHPs before and after controlled blood loss. Each group of NHPs (n=2) had 10%, 14%, 18%, 22%, or 25% of their total blood volume bled. The ratios to baseline levels were plotted: solid and dotted lines represent males and females, respectively. The levels at each time point were compared to the baseline levels and the difference was analyzed using the Wilcoxon paired test. D0AB, day 0 after bleeding; CK, creatine phosphokinase; AST, aspartate aminotransferase; ALT, alanine aminotransferase; TIBC, total iron binding capacity; UIBC, unsaturated iron binding capacity; GGT, gamma glutamyl transpeptidase; *P < 0.05; **P < 0.01.

The ratios of biological parameters at each time point compared to the baseline level are depicted in **Figure 4**. Glucose levels on D0AB and D1 were significantly higher than the baseline and normalized by D3. Albumin and total protein showed similar patterns of change. Their levels decreased on D0AB, reached a nadir on D1 or D3, and then began to increase. The levels of albumin and total protein from D0AB to D3 were significantly lower (*P* < 0.01), but total protein levels on D7 and D21 were higher than the basal levels (*P* < 0.05). The CK and AST levels significantly increased on D0AB and D1, peaking on D1. ALT levels significantly rose on D1 and D3, though their peak times varied. Creatinine levels increased on D0AB, while the BUN levels were variable. The iron levels significantly decreased after bleeding, remaining lower than the baseline value until D28. Accordingly, the TIBC and UIBC initially decreased but began to rise on D1 or D3, with their values on D7, D14, D21, and D28 being significantly higher than those at the baseline (*P* < 0.01).

#### 3.2.4 Changes in levels of immunological mediator

**Figure 5:**
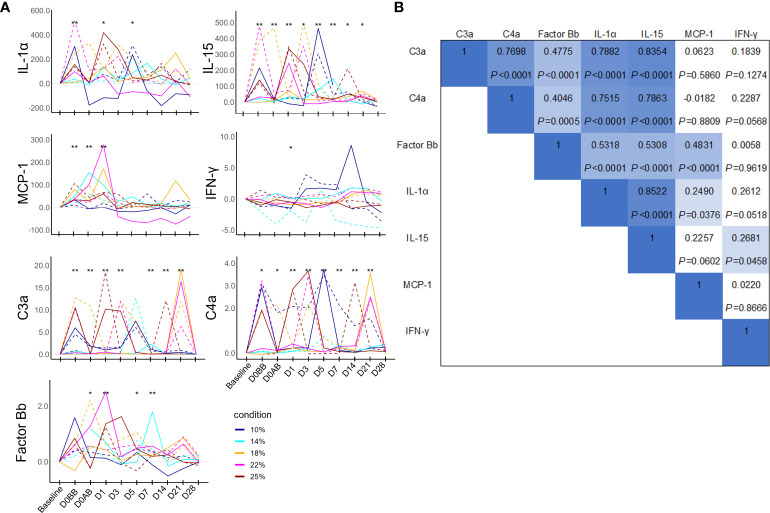
Humoral mediator responses in NHPs before and after controlled blood loss. Each group of NHPs (n=2) had 10%, 14%, 18%, 22%, or 25% of their total blood volume bled. **(A)** After subtracting baseline levels, the net values were plotted: solid and dotted lines represent males and females, respectively. The levels at each time point were compared to the baseline levels and the difference was analyzed using the Wilcoxon paired test. *P < 0.05; **P < 0.01. **(B)** The rho and *P* values of Spearman correlations between pairs of humoral mediators. IL, interleukin; MCP-1, monocyte chemoattractant protein-1; IFN, interferon.

Because the baseline levels of immunological parameters were low, individual changes in the immunological parameters were analyzed using the net values of each parameter by subtracting the baseline values to avoid analysis distortion (**Figure 5A**). The levels of IL-1β, IL-6, IL-8, IL-10, IL-12p70, and tumor necrosis factor were anecdotally detected but were mostly under the detection limits. Thus, these parameters were excluded from the analysis. The levels of immunological mediators demonstrated significant increases frequently during the experiment; however, pinpointing the exact timing of these increases proved challenging due to their variable nature. The data revealed that on D0BB, the levels of IL-1α, IL-15, C3a, C4a, and MCP1 were significantly elevated compared to the baseline, independent of blood loss effects. On D1, IL-15, C3a, factor Bb, and MCP-1 also exhibited a significant increase from the baseline levels. Conversely, IFN-γ levels did not show significant variation over time. Further, a correlation analysis of these immunological parameters (as depicted in **Figure 5B**) indicated a strong interrelationship between IL-15, IL-1α, C3a, and C4a, while factor Bb demonstrated a moderate correlation with these parameters.

### 3.3 Biological changes in response to blood loss

To investigate the influence of varying degrees of blood loss on biological responses in NHP subjects, we analyzed the relationship between the relative values (either ratio or difference) of parameters that differed significantly from baseline (*P* < 0.01) and the amount of blood loss. The findings are summarized in **Table 4**. The change in systolic BP level did not correlate with the extent of blood loss. In NHPs that experienced 25% blood loss and developed related symptoms, the systolic BP ratio on D0AB was significantly lower in this group compared to that in the others (median 0.65 vs 0.88, *P* = 0.0444).

**Table 4:** The Spearman correlation of the relative values to the amount of blood loss.

Variable	Time point	Rho	*P*		Variable	Time point	Rho	*P*	
Systolic BP	D0AB	-0.4182	0.2324		Glucose	D0AB	0.3333	0.3488	
RBC	D0AB	-0.7455	0.0184	*		D1	-0.3212	0.3677	
	D1	-0.8545	0.0035	**	Albumin	D0AB	-0.7576	0.0159	*
	D3	-0.7455	0.0184	*		D1	0.1636	0.6567	
	D5	-0.7818	0.0117	*		D3	-0.0424	0.9186	
	D7	-0.8303	0.0056	**		D5	-0.2121	0.5599	
	D14	-0.5030	0.1434		Total protein	D0AB	-0.6727	0.0394	*
Hemoglobin	D0AB	-0.7818	0.0117	*		D1	0.1273	0.7329	
	D1	-0.8788	0.0020	**		D3	0.2485	0.4916	
	D3	-0.6727	0.0394	*	CK	D0AB	0.4667	0.1782	
	D5	-0.7697	0.0137	*		D1	0.6485	0.0490	*
	D7	-0.8545	0.0035	**	BUN	D0AB	0.2970	0.4070	
	D14	-0.6485	0.0490	*		D1	-0.2242	0.5367	
Hematocrit	D0AB	-0.5758	0.0878		Creatinine	D0AB	0.6121	0.0665	
	D1	-0.6848	0.0351	*	AST	D0AB	0.2606	0.4697	
	D3	-0.6485	0.0490	*		D1	0.4667	0.1782	
	D5	-0.6364	0.0544		ALT	D1	0.5758	0.0878	
	D7	-0.8667	0.0027	**		D3	0.3576	0.3128	
MCV	D1	0.5273	0.1228			D7	0.1515	0.6818	
	D3	0.0545	0.8916		ALP	D0AB	-0.6970	0.0311	*
	D5	0.1879	0.6076			D1	0.3091	0.3871	
	D7	0.1515	0.6818		GGT	D0AB	-0.8182	0.0068	**
	D14	0.2364	0.5139			D1	-0.3576	0.3128	
	D21	0.0424	0.9186			D3	-0.0667	0.8648	
	D28	0.3697	0.2956			D5	-0.3576	0.3128	
MCH	D1	0.0182	0.9728			D7	-0.3091	0.3871	
	D14	-0.0182	0.9728		Cholesterol	D0AB	-0.6970	0.0311	*
MCHC	D5	-0.7939	0.0098	**		D1	-0.2485	0.4916	
	D14	-0.6485	0.0490	*		D7	0.1758	0.6320	
Reticulocyte	D3	-0.1515	0.6818		Triglyceride	D5	-0.5636	0.0958	
	D5	0.7818	0.0117	*	Calcium	D1	0.0545	0.8916	
	D7	0.7576	0.0159	*		D3	0.1394	0.7072	
	D14	0.3455	0.3305			D5	-0.2364	0.5139	
	D21	0.1879	0.6076		Iron	D3	-0.0909	0.8114	**
WBC	D0AB	-0.0545	0.8916			D5	-0.2848	0.4274	
Neutrophil	D0AB	-0.0182	0.9728			D7	-0.2606	0.4697	
Lymphocyte	D1	-0.2727	0.4483			D14	-0.8182	0.0068	**
	D3	-0.2606	0.4697		TIBC	D0AB	-0.8303	0.0056	**
	D5	-0.5152	0.1328			D1	0.0667	0.8648	
Monocyte	D0AB	-0.7818	0.0117	*		D7	0.6121	0.0665	
	D1	0.2606	0.4697			D14	0.5758	0.0878	
Platelet	D1	-0.7818	0.0117	*		D21	-0.7697	0.0137	*
	D5	0.4545	0.1909			D28	0.8909	0.0014	**
	D7	0.4182	0.2324		UIBC	D5	0.8061	0.0082	**
	D14	0.5152	0.1328			D7	0.6970	0.0311	*
	D21	0.1636	0.6567			D21	0.7333	0.0212	*
C3a	D0BB	0.1723	0.6340			D28	0.6727	0.0394	*
	D0AB	-0.8124	0.0043	**	Factor Bb	D1	0.7385	0.0147	*
	D1	0.1969	0.5855			D7	0.2216	0.5384	
	D14	-0.2462	0.4929		IL-15	D0BB	0.0985	0.7867	
	D21	-0.0246	0.9462			D0AB	0.1231	0.7348	
	D3	0.1231	0.7348			D1	0.8616	0.0014	**
	D7	-0.9109	0.0002	**		D5	-0.7385	0.0147	*
C4a	D1	0.6155	0.0582			D7	-0.2708	0.4492	
	D14	0.1477	0.6838		IL-1α	D0BB	0.1231	0.7348	
	D21	-0.0739	0.8393		MCP-1	D0AB	0.3693	0.2936	
	D3	0.0000	1.0000			D0BB	0.5416	0.1059	
	D5	-0.7139	0.0204	*		D1	0.5662	0.0879	
	D7	-0.5170	0.1260						

ALP, alkaline phosphatase; ALT, alanine aminotransferase; AST, aspartate aminotransferase; BP, blood pressure; BUN, blood urea nitrogen; CK, creatine phosphokinase; D0AB, day 0 after bleeding; D0BB, day 0 before bleeding; GGT, gamma glutamyl transpeptidase; IL, interleukin; MCH, mean corpuscular hemoglobin; MCHC, mean corpuscular hemoglobin concentration; MCP-1, monocyte chemoattractant protein-1; MCV, mean corpuscular volume; RBC, red blood cells; TIBC, total iron-binding capacity; UIBC, unsaturated iron-binding capacity; WBC, White blood cell; *P < 0.05; **P < 0.01.

Among hematological parameters, RBC-related measures including RBC, hemoglobin, hematocrit, MCHC, and reticulocyte counts correlated with the amount of blood loss. However, WBC and platelet counts (except on D1) did not show a relationship with blood loss. Biochemically, the ratios of albumin, total protein, ALP, GGT, total cholesterol, and TIBC on D0AB negatively correlated with blood loss, indicating a dilution effect due to blood loss. Conversely, increases in AST and ALT levels on D0AB and/or D1 were not related to blood loss. A positive correlation was observed between the increase in CK levels on D1 and the extent of blood loss. Analysis of the relationship between AST, ALT, and CK ratio values on D1 and the hemoglobin ratio on D1 revealed strong correlations (rho = -0.770, -0.818, and -0.915; *P* = 0.0092, 0.0038, and 0.0002, respectively), suggesting that a decrease in hemoglobin levels could trigger the responses of these enzymes.

Interestingly, the iron levels, which significantly decreased after blood loss, showed a strong negative correlation with the blood loss on D14. UIBC ratios on D5, D7, D21, and D28 positively correlated with blood loss, and TIBC ratios negatively correlated on D0AB but positively correlated with the blood loss on D21 and D28. Among the immunological parameters, net C3a values on D0AB and D7 negatively correlated, and net IL-15 values on D1 and D5 positively and negatively correlated, respectively, with the extent of blood loss, indicating high variability.

## 4 Discussion

Massive bleeding is a serious pathological condition that can lead to death without blood transfusion, making it an excellent disease model for developing RBC substitutes for blood transfusion. Investigating the therapeutic effect of RBC substitutes for blood transfusion warrants data on temporal changes in individual vitals for a simple blood loss situation. In clinical situations, massive bleeding may be associated with complex factors, such as trauma, underlying diseases, and treatment choices. Severe injury with massive bleeding has been linked to systemic inflammatory response syndrome, which is accompanied by a compensatory anti-inflammatory response characterized by elevated levels of anti-inflammatory cytokines, such as IL-10 and transforming growth factor-β, and cytokine antagonists, such as IL-1Ra (12).

Examining biological responses based on the degree of blood loss in primates, including humans, presents a considerable challenge owing to ethical issues. Considering *in vivo* experiments, various factors can impact subjects utilized when assessing the efficacy of blood substitutes. Our first step was to establish a blood-loss NHP model and examine the changes in hematological, biochemical, and immunological markers during blood loss. Leukocyte-related changes, especially, increased levels of neutrophils and proinflammatory cytokines (13), which are commonly seen in patients receiving blood, occurred immediately after catheterization in NHPs and were not directly related to the amount of blood loss. Subsequently, we systematically tested individual factors in a clinical situation of complex massive blood loss to explore direct biological changes.

The Advanced Trauma Life Support (ATLS) Manual provided by the American College of Surgeons, describes four classes of hemorrhage to emphasize early signs of shock (14). Based on the total blood volume, class I hemorrhage is classified as a loss of up to 15%, class II hemorrhage as 15 to 30%, class III hemorrhage is classified as a loss of 30 to 40%, and class IV hemorrhage is classified as a loss of > 40%. Symptoms of class II hemorrhage include tachycardia, tachypnea, and decreased pulse pressure. There may be a sense of coolness and clamminess on the skin as well as a delay in the degree of capillary refill. Changes in mental status are presented with class III hemorrhage. According to the ATLS manual, monitoring is recommended in cases of class I hemorrhage, and blood transfusion is recommended in cases of class II hemorrhage. In clinical practice, a transfusion trigger, based on the hemoglobin level, is used when deciding on blood transfusion (15). However, no criteria have been established for blood loss in NHPs. In this investigation, clinical symptoms of decompensation and a substantial decrease in systolic BP were observed exclusively in NHPs subjected to 25% blood loss, yet this did not result in any fatalities. Consequently, we postulate that a blood loss volume of 25% is an appropriate threshold in NHP models for assessing the efficacy of blood transfusion or alternative therapeutic interventions. Regarding blood donation protocols, it is advisable that the volume donated should not exceed 25% of the total blood volume in NHP donors to avoid inducing physiological decompensation, as evidenced by the consequences of a 25% blood loss.

RBC-related parameters demonstrated the most direct correlation with blood loss. In the ratio plots against baseline results, RBC, hemoglobin, and hematocrit exhibited nearly identical trends, reaching their nadir between D1 and D5. The lowest hemoglobin level, typically observed between 1–3 days after bleeding, was likely due to the redistribution of body fluids. This value varied between D1–D5 for each individual, with precise timing challenging to ascertain due to the insufficient frequency of blood collection. Reticulocyte counts began to rise from D3, peaking at D7 across all subjects, followed by normalization. The increase in MCV after bleeding is presumed to result from an elevated production of comparatively larger young RBCs; the bleeding increases their proportion relative to that of old RBCs with low MCV. In this study, analyzing the data as ratios to baseline proved more useful due to significant individual variation. Most soluble substances in the blood experienced a temporary dilution due to blood loss before recovery.

The levels of some enzymes, such as CK, AST, and ALT, showed reactive increases, which are hypothesized to be a complex effect of blood loss and experimental injury. The increased ratios of the levels of these enzymes after bleeding correlated more strongly with the hemoglobin ratio than with the amount of blood loss, suggesting that the hemoglobin ratio value might be a more reliable indicator for predicting clinical state than the quantity of blood loss itself. Previous human studies have detected no or only weak correlations between estimated blood loss during surgery and changes in hemoglobin (16, 17). Given that intraoperative blood loss is estimated visually, it cannot be empirically correlated with changes in hemoglobin levels. Elevated liver enzyme levels have been reported in various situations, and aminotransferases have been found to increase by 10- to 50-fold the upper limit in ischemic injuries. The increase in liver enzyme levels observed in our study appears to align with findings from previous research, indicating that acute massive bleeding leads to instantaneous tissue ischemia and elevated liver enzyme levels (18).

Blood loss led to a decrease in serum iron levels, which reached their lowest between 5–7 days after bleeding before gradually recovering; however, levels had not normalized by day 28. Notably, both TIBC and UIBC continued to rise even on day 28, indicating unresolved iron deficiency. While this study could not provide a definitive interval for blood donation, the results suggest that after a 25% blood loss, a one-month recovery period before subsequent donations might not be sufficient; iron supplementation should be considered if necessary.

The levels of IL-1α, IL-15, and MCP-1, representative proinflammatory cytokines, increased from D0BB (before bleeding), suggesting that these changes were related to the experimental process rather than the bleeding itself. This observation was similar in the increase in the WBC and neutrophil counts. The levels of the complement activation fragment C3a and C4a, which are also associated with these inflammatory cytokines, showed increases and were sometimes negatively correlated with the amount of blood loss. This may be due to a combined effect of inflammatory responses and the reduced concentrations of the C3 and C4 proteins due to blood loss.

Accumulated evidence on postoperative bleeding has shown that surgical trauma can considerably impact a patient’s immune system. Intraoperative blood loss during major surgery leads to reduced blood flow, regional hypoxia, and metabolic and microenvironmental changes, resulting in an inflammatory response characterized by proinflammatory cytokines and acute phase proteins (19). Similarly, during surgery, there is an early hyperinflammatory response involving the release of proinflammatory cytokines, such as tumor necrosis factor-alpha, IL-1, and IL-6, neutrophil activation, microvascular adhesion, as well as an uncontrolled oxidative burst of polymorphonuclear cells and macrophages (20). In previous studies, various factors that could affect the patient’s condition were not controlled owing to the surgical environment, and the precise amount of bleeding could not be measured. In the current study, we strictly controlled the amount of bleeding from 10 to 25%, depending on the body weight of the NHPs, illustrating the strength of our study.

A major limitation of our study was the small number of NHPs assigned to each condition. Although a difference was observed in the results for each condition, it is unclear whether this reflects a difference in the baseline of the individual or condition. To overcome these limitations, we statistically analyzed the increase (ratio) or change (differences) in each individual, based on their baseline values. Increasing the number of NHPs in each condition would be statistically beneficial but difficult in practice. This is a general limitation of NHP research. A previous NHP research review has shown that the number of individuals assigned to groups ranges from 1 to 8 for the smallest group size and 3 to 16 for the largest group size (21). In our NHP-based experiment, we used the relative values (ratio or difference) compared to the baseline values for the statistical analysis to minimize the impact of the limitation attributed to the small number of animals.

The implications of our study extend to predictive medicine, especially regarding the biological responses during massive blood loss. Controlled experiments on humans in emergencies, such as massive bleeding, often encounter ethical and practical constraints. Thus, our study provides a useful framework to design primate experiments on blood loss or transfusion, particularly when considering intervention effects following blood loss, and can aid in interpreting the results of related primate experiments. For future studies, we are planning a xenotransfusion experiment using porcine erythrocytes to analyze their biological effects in primates.

## Data availability statement

The original contributions presented in the study are included in the article/supplementary material. Further inquiries can be directed to the corresponding author.

## Ethics statement

The animal study was approved by Korea Institute of Toxicology Institutional Animal Care and Use Committee (IAC-22-01-0342-0037). The study was conducted in accordance with the local legislation and institutional requirements.

## Author contributions

JR: Formal analysis, Visualization, Writing – original draft. EP: Data curation, Investigation, Methodology, Writing – review & editing. HL: Data curation, Investigation, Methodology, Writing – review & editing. JH: Project administration, Supervision, Writing – review & editing. H-SK: Data curation, Investigation, Methodology, Writing – review & editing. JP: Formal analysis, Software, Writing – review & editing. HJK: Writing – original draft, Conceptualization, Funding acquisition, Project administration, Resources, Supervision.
